# Editorial: Neurotechnology for brain-body performance and health: insights from the 2022 Neuroergonomics and NYC Neuromodulation Conference

**DOI:** 10.3389/fnrgo.2024.1454889

**Published:** 2024-09-03

**Authors:** Marom Bikson, Leigh Charvet, Giuseppina Pilloni, Frederic Dehais, Hasan Ayaz

**Affiliations:** ^1^Department of Biomedical Engineering, The City College of New York, The City University of New York, New York, NY, United States; ^2^Department of Neurology, NYU Grossman School of Medicine, New York, NY, United States; ^3^ISAE-SUPAERO, Université Fédérale de Toulouse, Toulouse, France; ^4^School of Biomedical Engineering Science and Health Systems, Drexel University, Philadelphia, PA, United States

**Keywords:** neurotechnology, neuroergonomics, neuromodulation, electroencephalography (EEG), functional near infrared spectroscopy (fNIRS), transcranial direct current stimulation (tDCS), neurostimulation

## Introduction

Neuroergonomics is an emerging interdisciplinary field that aims to understand and utilize brain function during real-world tasks in everyday life, for both healthy and clinical conditions (Parasuraman, 2003; Ayaz and Dehais, 2019). Neuroergonomics combines multidisciplinary approaches and methods to explore new scientific frontiers in human brain function research and applications (Dehais et al., 2020).

This Research Topic invited submissions exploring novel approaches and emerging directions in neuroergonomics to advance our understanding of neurophysiological measures and their relationship to complex tasks. The contributions are expected to address a range of Research Topics, including non-invasive neuroimaging and brain stimulation techniques designed to enhance human performance, mitigate disease burden, and deepen our comprehension of complex brain functions.

Submissions were encouraged to focus on advanced neuroscience and neuroengineering methods, alongside neurostimulation and neuroimaging technologies, to investigate brain dynamics in actively behaving participants within real or realistic field settings. This includes the application of these technologies to study various cognitive and emotional processes such as perception, decision-making, attention, working memory, and cognitive workload. Additionally, performance monitoring, human-machine interaction, brain-computer interfaces, mobile brain and body imaging, neuroadaptive technologies, and related areas relevant to working environments were also areas of particular interest.

By integrating these diverse yet interconnected research areas, the Research Topic aims to foster innovation and collaboration in the field of neuroergonomics, paving the way for practical applications and theoretical advancements that can enhance our understanding of the human brain in complex, real-world contexts.

There were three publications in this Research Topic in addition to the four publications in the parallel Research Topic (Neurotechnology for sensing the brain out of the lab: methods and applications for mobile functional neuroimaging), and included neuromodulation, specifically transcranial direct current stimulation (tDCS) that can be used as a therapeutic tool to enhance function and alleviate symptoms in various central and peripheral nervous system disorders (Bikson et al., 2020). A critical advantage of tDCS among non-invasive brain stimulation techniques is the potential for wearability and portability, enabling administration outside of clinical and research settings. This allows for supervised self-administration in-home environments, broadly expanding its deployability and scalability for research and treatment applications (Charvet et al., 2020).

In the first contribution to the Research Topic, Schwertfeger et al. reported a mini-review of transcranial direct current stimulation (tDCS) studies aimed at improving cognition in adults with traumatic brain injury (TBI). As a tDCS application, authors reviewed a total of 399 results and found that 12 of them met the criteria for inclusion. These results represent research from Australia, Canada, Italy, South Korea, Poland, and the USA, and were published between 2012 and 2021. They discussed various categories of research, including studies of people with chronic TBI (more than 6 months post-TBI), studies of people undergoing TBI rehabilitation (< 6 months post-TBI), studies using a single tDCS session and multiple sessions, with and without a behavioral intervention. They showed promising results from limited studies for each TBI acuity and severity level, and these support the need for further studies.

The Research Topic was also focused on wearable neuroimaging, both electroencephalography (EEG), and functional near infrared spectroscopy (fNIRS). For a recent comprehensive review of fNIRS methodology and applications, see Ayaz et al. (2022), and for a discussion of emerging EEG methods, see Gramann et al. (2014). The remaining contributions focus on individual and multi-modal use of these brain sensing techniques.

The second contribution to the Research Topic was by Kounios et al., and the study reported an estimation of brain age using a low-cost EEG headset. In this study, authors described an EEG-based machine-learning technology for assessing whether an individual's brain is aging more quickly or more slowly than would be expected relative to healthy individuals of the same age. Understanding and measuring brain age is crucial for identifying individuals at risk of diseases or cognitive decline, enabling timely examinations to detect and diagnose disorders that become harder to treat as they progress. It also helps in assessing how neurological disorders, injuries, and environmental factors can accelerate brain aging, as well as how specific lifestyles might help preserve or enhance brain health. In this novel study, authors developed and tested a machine learning model to predict individual participants' brain ages based on data recorded with a low-cost EEG headset. The authors concluded that reliable estimates of a person's brain age could be made from only 12 min of resting-state EEG.

The final contribution to the Research Topic was by Mark et al., who reported mental workload assessment by monitoring the brain, heart, and eye with six biomedical modalities during six cognitive tasks. Mental workload (MW) is a core concept in neuroergonomics, and an accurate assessment of MW could help prevent human operator errors during the performance of complex tasks and allow for pertinent intervention by predicting performance declines that can arise from either work overload or under-stimulation. In this study, authors developed a new six-cognitive-domain task protocol, coupling it with six biomedical monitoring modalities to concurrently capture performance and cognitive workload correlates across a longitudinal multi-day investigation. Authors utilized two distinct modalities for each aspect of cardiac activity, ocular activity, and brain activity (EEG and fNIRS), with participants engaged in four sessions over 4 weeks, performing tasks associated with working memory, vigilance, risk assessment, shifting attention, situation awareness, and inhibitory control. This is the first comprehensive comparison of these six brain-body measures across multiple days and cognitive domains. The findings underscore the potential of wearable brain and body sensing methods for evaluating mental workload. Such comprehensive neuroergonomic assessment can inform the development of next-generation neuroadaptive interfaces and training approaches for more efficient human-machine interaction and operator skill acquisition.

## Report from the 2022 joint meeting of Neuroergonomics Conference and NYC Neuromodulation Conference

On July 28 to August 1, 2022, 100's of neural engineers, cognitive neuroscientists, biomedical engineers, neuroergonomists, and brain technologists came together in New York City for the 2022 joint meeting of the Neuroergonomics Conference & NYC Neuromodulation Conference. This was the 4th iteration of the Neuroergonomics Conference (previously held in 2016, 2018, and 2021), the 6th iteration of the NYC Neuromodulation Conference (previously held in 2013, 2015, 2018, 2019, and 2020), and marked the first time the two conference we run jointly. The scientific committee was Dr. Leigh Charvet (Conference Co-Chair), Dr. Marom Bikson (Conference Co-Chair), Dr. Giuseppina Pilloni (Technical Program Chair), Dr. Frédéric Dehais (Steering Committee), Dr. Hasan Ayaz (Steering Committee), Dr. Roy Hamilton, Dr. Evangelia Chrysikou, Dr. Tracy Dennis, and Dr. Ranjana K Mehta, and held at the historic campus of The City College of New York. This Research Topics of Frontiers in Neuroergonomics features articles building on content presented at the conference.

For the 2022 meeting, Neuroergonomics and NYC Neuromodulation Conferences joined together to address the state-of-the-art in neurotechnology for brain-body performance and health. Neurotechnology represented at the conference spanned the extremes. From critical care, to wellbeing, to the brain in every-day life. From revolutionary invasive devices, to targeted non-invasive approaches, to wearables. From boosting the performance of athletes, surgeons, artists, first responders, and to service members. From brain-to-brain interfaces to mixed/virtual reality to social media. The conference focused on the latest approaches for both brain function and dysfunction including brain/body performance, skill acquisition, stress and fatigue, pain, addiction and binge eating, cognition and physical recovery, eye-tracking, neuromarketing, and remote/mobile sensing in the wild. These themes are intended to encouraged discussion that crosses traditional sub-domains of brain and health technologies.

Highlights from the conference includes talk by Dr. Ali Rezai on “Neuromodulation for addiction,” Dr. Andrew Stephen Huhn on “Wearable neuroimaging for opioid use disorder treatment,” Dr. Barbara Colombo on “Cognitive reserve, aging, emotion, creativity and brain stimulation,” Dr. Rebecca Jones on “Detection of eye contact with deep neural networks is as accurate as human experts,” Dr. Daniel Callan on “Measuring and stimulating the brain at the extremes of performance,” Dr. Inês Violante on “Targeting the Human Hippocampus with Non-Invasive Deep Brain Stimulation,” Dr. Faranak Farzan on “EEG microstates, fluid intelligence, cognitive training, and brain stimulation,” Dr. Maria Schultheis on “The application of virtual reality technology in rehabilitation,” Dr. Robert Jacob on “fNIRS as an input to brain computer interfaces,” Dr. Flavia Vitale on “MXene-infused bioelectronic interfaces for multiscale electrophysiology and stimulation,” Dr. Mary Lou Jepsen on “Next-gen portable neuroimaging for medical diagnostics,” Dr. Uri Hasson on “Shared computational principles for language processing in humans and deep language models,” Dr. Riki Banerjee on “Endovascular brain computer interface,” Dr. Cristin Grace Welle on “VNS enhances motor learning and myelin plasticity,” and dozens of additional talks.

The following iteration of the Neuroergonomics Conference is scheduled for July 8–12, 2024, in Bordeaux, France, and the following NYC Neuromodulation Conference is scheduled for August 1–3, 2024, in New York City.

## References

[B1] Ayaz H. Baker W. B. Blaney G. Boas D. A. Bortfeld H. Brady K.. (2022). Optical imaging and spectroscopy for the study of the human brain: status report. Neurophotonics 9:S24001. 10.1117/1.NPh.9.S2.S24001 36052058 PMC9424749

[B2] Ayaz H. Dehais F. (2019). Neuroergonomics: The Brain at Work and Everyday Life, 1st Edn. Elsevier Academic Press. Available at: https://www.elsevier.com/books/neuroergonomics/ayaz/978-0-12-811926-6

[B3] Bikson M. Hanlon C. A. Woods A. J. Gillick B. T. Charvet L. Lamm C.. (2020). Guidelines for TMS/tES clinical services and research through the COVID-19 pandemic. Brain Stimul. 13, 1124–1149. 10.1016/j.brs.2020.05.010 32413554 PMC7217075

[B4] Charvet L. E. Shaw M. T. Bikson M. Woods A. J. Knotkova H. (2020). Supervised transcranial direct current stimulation (tDCS) at home: a guide for clinical research and practice. Brain Stimulat. 13, 686–693. 10.1016/j.brs.2020.02.011 32289698

[B5] Dehais F. Karwowski W. Ayaz H. (2020). Brain at work and in everyday life as the next frontier: grand field challenges for neuroergonomics. Front. Neuroergonom. 1:583733. 10.3389/fnrgo.2020.583733 38234310 PMC10790928

[B6] Gramann K. Ferris D. P. Gwin J. Makeig S. (2014). Imaging natural cognition in action. Int. J. Psychophysiol. 91, 22–29. 10.1016/j.ijpsycho.2013.09.003 24076470 PMC3983402

[B7] Parasuraman R. (2003). Neuroergonomics: research and practice. Theoret. Iss. Ergon. Sci. 4, 5–20. 10.1080/14639220210199753

